# Investigations on the Antifungal Effect of Nerol against *Aspergillus flavus* Causing Food Spoilage

**DOI:** 10.1155/2013/230795

**Published:** 2013-12-23

**Authors:** Jun Tian, Xiaobin Zeng, Hong Zeng, Zhaozhong Feng, Xiangmin Miao, Xue Peng

**Affiliations:** ^1^College of Life Science, Jiangsu Normal University, Xuzhou, Jiangsu 221116, China; ^2^Guangdong Key Laboratory for Research and Development of Natural Drugs, Department of Pharmacology, Guangdong Medical College, Zhanjiang, Guangdong 524023, China; ^3^Key Laboratory of Protection and Utilization of Biological Resources, Tarim University, Alar, Xinjiang 843300, China

## Abstract

The antifungal efficacy of nerol (NEL) has been proved against *Aspergillus flavus* by using *in vitro* and *in vivo* tests. The mycelial growth of *A. flavus* was completely inhibited at concentrations of 0.8 **μ**L/mL and 0.1 **μ**L/mL NEL in the air at contact and vapor conditions, respectively. The NEL also had an evident inhibitory effect on spore germination in *A. flavus* along with NEL concentration as well as time-dependent kinetic inhibition. The NEL presented noticeable inhibition on dry mycelium weight and synthesis of aflatoxin B_1_ (AFB_1_) by *A. flavus*, totally restraining AFB_1_ production at 0.6 **μ**L/mL. In real food system, the efficacy of the NEL on resistance to decay development in cherry tomatoes was investigated *in vivo* by exposing inoculated and control fruit groups to NEL vapor at different concentration. NEL vapors at 0.1 **μ**L/mL air concentration significantly reduced artificially contaminated *A. flavus* and a broad spectrum of fungal microbiota. Results obtained from presented study showed that the NEL had a great antifungal activity and could be considered as a benefit and safe tool to control food spoilage.

## 1. Introduction

Fungi, especially *Aspergillus flavus*, are able to contaminate many kinds of food including fruit, vegetables, and cereals. *A. flavus* is the most common species mainly involved in food spoilage in particular by secretion of highly poisonous secondary metabolites—aflatoxin. Aflatoxin is the most toxic form for mammals and presents carcinogenic, teratogenic, hepatotoxic, mutagenic, and immunosuppressive properties and can inhibit several aspects of the metabolic system [1]. So, there is a need for safe and effective methods to control contamination of *A. flavus* of food.

Widespread public concern for health and environmental effects of synthetic fungicides and the restriction of their use have led to an urgent push for the search for safer alternative natural preservatives to replace synthetic chemicals. The antimicrobial properties of plant products have been well known and used for food preservation and in medicine for centuries. Nowadays, interest in natural antimicrobial compounds as food preservation and medicine has been increasingly growing.

**Figure 1:**
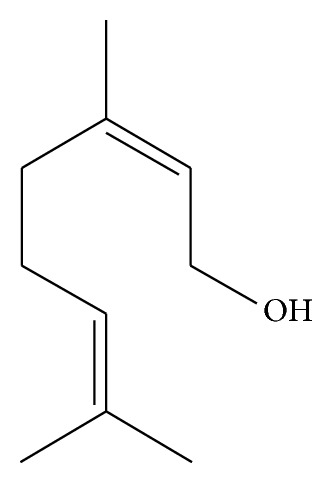
Structure of NEL.

Nerol (NEL), a kind of monoterpene compound (Figure 1), was originally isolated from neroli oil and also found in many essential oils such as rose and lavender [2, 3]. For their especially pleasant sweet-floral impressions, NEL is used in flavors in food products, such as nonalcoholic beverages, candies, puddings, ice creams, baked goods, chewing gums and syrups [4]. NEL is also widely used in decorative cosmetics, fine fragrances, shampoos, toilet soaps, and other toiletries as well as household cleaners and detergents [5]. NEL are known to have antimicrobial activities against some gram-(positive) and gram-(negative) bacteria, as well as the yeast *Candida albicans* [4]. To date, to our knowledge and according to the literature survey, there are no available reports on the antifungal activity *in vitro* and *in vivo* of NEL to control food spoilage.

In this investigation, NEL was evaluated for its effects on the mycelial growth, spore germination, mycelium weight, and aflatoxin B_1_ (AFB_1_) content in *A. flavus*. Moreover, the potential application of the NEL to control postharvest spoilage on stored cherry tomatoes was also assessed.

## 2. Material and Methods

### 2.1. Chemicals

NEL (97.0%) used in this investigation was purchased from Aladdin Chemical Reagent Co., Ltd. (Shanghai, China) and it was prepared as a stock solution in 5% (v/v) Tween-20 and kept at approximately 4°C in dark condition for the study.

### 2.2. Microorganism and Media

*Aspergillus flavus* CCAM 080001 was obtained from the Culture Collection of State Key Laboratory of Agricultural Microbiology (CCAM), China. The fungal strain cultures were maintained on a potato dextrose agar (PDA) slant at 4°C in a refrigerator. The old cultures were transferred to fresh slope every two months to avoid a decline in strain viability.

### 2.3. Effect of NEL on Mycelial Growth

The antifungal activity of NEL was estimated by its contact and vapor phase effects on the mycelial growth of *A. flavus* with some modifications [6, 7]. For the determination of the contact phase effect of NEL, which inhibits hyphal growth, aliquots of the NEL dissolved separately in 0.5 mL of 5% (v/v) Tween-20 were pipetted aseptically onto glass petri dishes (9 × 1.5 cm) containing 9.5 mL PDA medium at a temperature of 45–50°C to procure the required concentrations of 0.1, 0.2, 0.4, 0.6, and 0.8 *μ*L/mL. Agar discs (9 mm diameter) from the edge of a 5-day old *A. flavus* culture were placed at the center of each Petri plate, which was sealed with parafilm. The controls did not contain NEL. Then, they were incubated at 28 ± 2°C.

In the vapor phase, 20 mL PDA was pipetted aseptically onto glass Petri dishes, and one disc (9 mm) from the edge of a 5-day old *A. flavus* culture was placed on PDA in each plate. The Petri plates were inverted, and NEL with 1 mL of different concentrations of NEL were dissolved in sterile 5% (v/v) Tween-20 were added to the 70 mm sterile blank filter disks and placed on the medium-free cover of each Petri plate to obtain final concentrations of 0.0125, 0.025, 0.05, 0.075, and 0.1 *μ*L/mL air. The Petri plates were then sealed using parafilm and inoculated as described above.

The rate of colony growth was measured based on its radius increasing. The effect of the treatment was determined each day for 9 days by measuring the average of 2 perpendicular diameters of each colony. In all cases, the experiments were carried out in triplicates. The inhibitory effect of NEL on the mycelial growth percentage, compared with the control group, was calculated at day 9, according to the following formula [8]:

(1) $$\begin{array}{c} \text{percentage}\;\text{mycelial}\;\text{inhibition}=\left[\frac{dc-dt}{dc}\right]\times 100, \end{array}$$

where *dc* (cm) is the mean radius of the colony in the control plates and *dt* (cm) is the mean colony radius value of colonies grown in PDA media containing NEL.

### 2.4. Effect of NEL on Spore Germination

Fungal spore germination and growth kinetics were assayed following our previously published method [9]. NEL was added to the glass tube containing 1 mL 0.1 (v/v) Tween-20 to obtain the final concentrations of 0.1, 0.2, 0.4, 0.6, and 0.8 *μ*L/mL. A spore suspension of *A. flavus* was prepared by adding 5 mL of sterile water containing 0.1% (v/v) Tween-20 to each Petri dish and gently flooding the surfaces of the 3-day-old fungal culture 3 times with a sterile L-shaped spreader to free spores. After adjustment of spores concentration (10^7^ spores/mL), the homogenous spore suspension of *A. flavus *was then transferred to each of the above tubes. From this, 10 *μ*L aliquots of the spore suspension were incubated into fresh PDA media in separate depression slides. Depression slides containing the spores were assembled with the cover slip and incubated in a moisture chamber at 28°C for 20 h in three replicates. The number of germinated and nongerminated spores was counted and the percentage of spore germination was calculated. About 200 single spores per treatment were examined. The spores were considered germinated when the germ-tube was longer than the spore diameter.

*A. flavus* was also examined in a kinetic study to assess its antifungal activity. Aliquots (10 *μ*L) of the spore suspension of *A. flavus* containing 10^8^ spores/mL prepared in 0.1% (v/v) Tween-20 was aseptically transferred to different concentrations (0.2, 0.4, and 0.8 *μ*L/mL) of 5 mL NEL solution, and a homogenous suspension (about 2 × 10^5^ spore/mL) was mixed vigorously by vortex (TS-1, Kylin-Bell Lab Instruments Co., Ltd., Shanghai, China) for 1 min. The controls were prepared similarly with the exception of the NEL treatment. After specific time intervals, that is, 30, 60, 90, 120, 150, and 180 min, the reaction mixtures were collected and centrifuged at 6000 ×g (TGL-16C, Anting Scientific Instrument Factory, Shanghai, China) for 5 min at room temperature. The supernatant was discarded and the remainder was resuspended in 10 mL of sterilized distilled water. Aliquots (10 *μ*L) of the spore suspension were taken to the depression slides, which were handled as described above. A total of 200 single spores per treatment were observed and the percentage of spore germination was calculated.

### 2.5. Effect of NEL on AFB_**1**_ Synthesis

The inhibitory effects ofNEL on AFB_1_-biosynthesis by *A. flavus* were investigated following Kumar et al. [10]. Aliquots (100 *μ*L) of the spore suspension of *A. flavus* containing 10^7^ spores/mL prepared in 0.1% (v/v) Tween-20 was added to 20 mL potato dextrose broth (PDB) medium in Erlenmeyer flasks. The required amounts of NEL dissolved in 5% (v/v) Tween-20 were added to the PDB medium to obtain 0.1, 0.2, 0.4, 0.6, and 0.8 *μ*L/mL concentrations. Samples without any NEL treatment were considered controls. The flasks were incubated at 28 ± 2°C for 10 days. Three replicates of each treatment were performed, and the experiment was repeated three times. After 10 days on rotary shaker, the mycelia produced in liquid cultures were filtered and washed through filter paper (DX102, Xinhua Paper Co., Ltd., Hangzhou, China). The weight of the dry mycelia for each mycelium was measured after drying them at 60°C for 24 h.

For extraction, detection, and quantification of AFB_1_, the filtrate was partitioned twice with 25 mL chloroform in a separating funnel. The chloroform extracts were combined and evaporated to dryness and the residue was resuspended in chloroform up to 1 mL in a volumetric flask. A silica gel-G thin layer plate was used for the AFB_1_ analysis. Aliquots (50 *μ*L) of each sample spotted onto the TLC sheets were developed in the solvent system comprising toluene : isoamyl alcohol : methanol (90 : 32 : 2 v/v/v) [11]. The identity of AFB_1_ was observed under a UV lamp at 365 nm and confirmed chemically by spraying trifluoroacetic acid [12]. For the quantification of AFB_1_, amethyst-fluorescent spots of AFB_1_ on the TLC were scraped out, dissolved in 5 mL cold methanol, and centrifuged at 2000 ×g (TGL-16C, Anting Scientific Instrument Factory, Shanghai, China) for 5 min. The absorbance of the supernatant was performed by UV-visible spectrophotometer (UV-2802, Unico Co. Ltd., Shanghai, China) at a wavelength of 360 nm. The AFB_1_ content present in the sample was calculated following a formula by Sinha et al. [13]:

(2) $$\begin{array}{c} {\text{AFB}}_{1}\;\text{content}\;\;\left(\mu \text{g}/\text{mL}\right)=\frac{\left(D\times M\right)}{\left(E\times l\right)}\times 1000, \end{array}$$

where *D* is the absorbance, *M* is the molecular weight of aflatoxin (312), *E* is the molar extinction coefficient (21, 800), and *l* is the path length (1 cm cell was used).

AFB_1_ inhibition was calculated as follows:

(3) inhibition  (%)=(1−XY)×100,

where *X* represents the mean concentration of AFB_1_ in the treatment and *Y* represents the mean concentration of AFB_1_ in the control.

### 2.6. Effects of NEL on Wound-Inoculated Cherry Tomatoes

Cherry tomato (*Lycopersicon esculentum*) fruits in commercial maturation stage were obtained from local market and selected for uniformity in size, appearance, ripeness, and the absence of physical defects. The fruits were divided into 3 replicates (12 fruits per replicate) and surfaced-disinfected with 70% ethanol for 2 min, rinsed twice with double distilled water (5 min each) and then left for 1 h in safe cabinet to dry. One uniform wound (4 mm diameter, 2 mm deep) was made per fruit using a sterilized cork borer. Each wound was separately inoculated with 10 *μ*L of a conidial suspension containing 1 × 10^6^ spores/mL of *A. flavus*. NEL with concentrations of 40, 80, 120, and 160 *μ*L/mL dissolved separately in 0.5 mL of 5% (v/v) Tween-20 was pipetted aseptically onto filter paper discs of 4 cm diameter and respectively placed into individual weighing bottles (*φ* 40 × 25 mm) without lids to obtain final concentrations of 0.025, 0.05, 0.075, and 0.1 *μ*L/mL air. At the same time, the filter paper moistened with sterilized water was placed in each container in order to maintain high-relative humidity (90–95%) during the storage period. Controls were prepared similarly with the exception of the volatile treatment. All the containers were then transferred to storage at 18°C for 9 days. At the end of storage, the percentage of infected fruits was measured. The entire experiment was conducted twice.

### 2.7. Effects of NEL on Cherry Tomatoes Decay

A random selection of cherry tomatoes without any handling were randomly distributed into three replicates (12 fruits per replicate) and then placed in 0.9 L polystyrene containers with snap-on lids. Four treatments (NEL concentrations of 0.025, 0.05, 0.075, and 0.1 *μ*L/mL air) and sterilized water were prepared as described above. Controls were treated with the same volume of sterile distilled water under the same conditions. The percentage of infected fruits was measured after 21 days at 18°C. All treatments consisted of 3 replicates with 12 fruits per replicate. The entire experiment was conducted in twice.

### 2.8. Sensory Evaluation

Sensory evaluation was carried out by using our previously published method with some modifications [14]. Twelve panelists scored the samples for overall acceptability in artificially inoculated groups at 3, 6, and 9 days of storage, and in naturally infected groups at 7, 14, and 21 days of storage. Panelists were also asked to provide additional qualitative comments for each sample. The panelists included 12 consumers (50% female and 50% male, ranging from 16 to 54 years old). A questionnaire was used to record the data. A 9-point hedonic scale (9 = like extremely; 8 = like very much; 7 = like moderately; 6 = like slightly; 5 = neither like nor dislike; 4 = dislike slightly; 3 = dislike moderately; 2 = dislike very much; 1 = dislike extremely) was used to score the samples.

### 2.9. Statistical Analysis

Experimental data were analyzed using SPSS 13.0 statistical software (Chicago, USA). The mean values were calculated and reported as the mean ± standard deviations (*n* = 3). The one-way analysis of variance procedure followed by Duncan's multiple range test at the *P* < 0.05 level was used to measure the significant difference of the dates.

## 3. Results 

### 3.1. Antifungal Effects of NEL on Mycelial Growth

**Figure 2:**
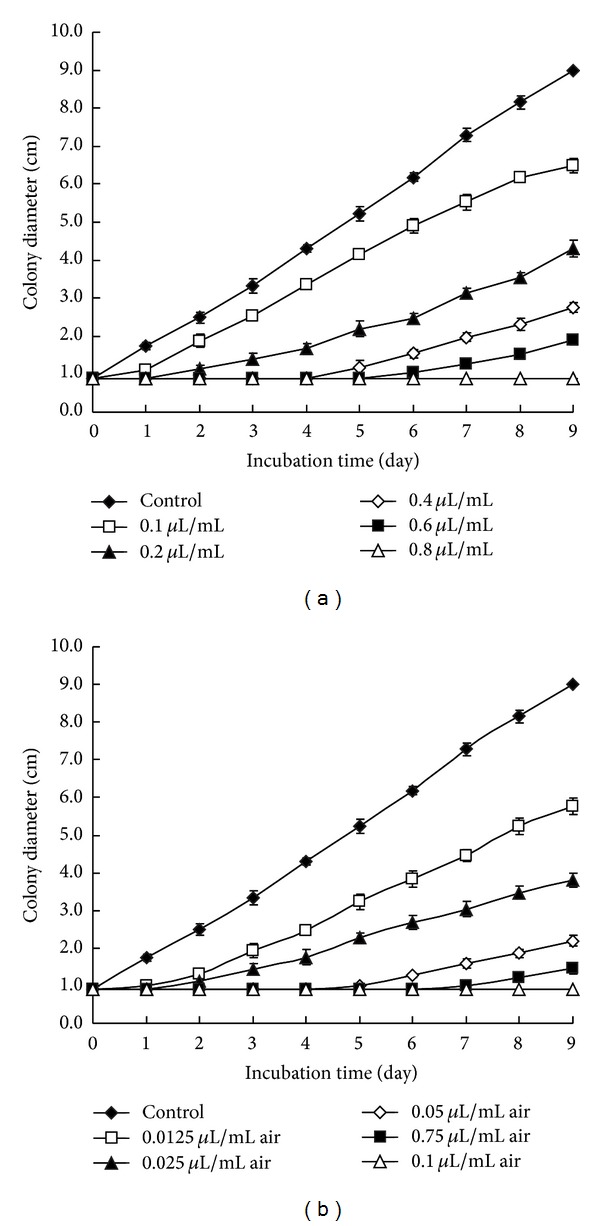
Effects of different concentrations of contact (a) and vapor (b) phase of NEL on colony growth of *A. flavus*. The plates were incubated at a temperature of 28 ± 2°C for 9 days. Values are means (*n* = 3) ± standard deviations.

The contact and vapor phase effects of different concentrations of NEL on the colony growth of *A. flavus in vitro* are showed in Figures 2(a), and 2(b). Results present that all concentrations of NEL decreased the fungal growth and mycelia growth was considerably reduced with the increasing concentration of NEL but it increased with incubation time. In contact phase testing, the colony growth was delayed by 5 days for *A. flavus* at 0.6 *μ*L/mL concentration; and the level of growth inhibition at the concentration of 0.8 *μ*L/mL was 100% and the growth was completely inhibited after 9 days of incubation (Figure 2(a)). The NEL produced a significant reduction in mycelium growth with *A. flavus* at 0.1, 0.2, 0.4, and 0.6 *μ*L/mL concentrations with reduction percentages of 31.0%, 58.1%, 77.0%, and 87.8%, respectively. Vapor phase effects of NEL were greater on mycelial growth than contact inhibitory effects were. Results of the vapor phase effect of NEL are indicated in Figure 2(b). In the vapor phase test at 0.1 *μ*L/mL NEL concentration in air, fungal development was totally inhibited after 9 days of incubation. A significant reduction was also observed in mycelium growth with *A. flavus* at 0.0125, 0.025, 0.05, and 0.075 *μ*L/mL air concentrations with reduction percentages of 39.9%, 64.1%, 84.0%, and 93.0%, respectively.

### 3.2. Antifungal Effects of NEL on Spore Germination

**Figure 3:**
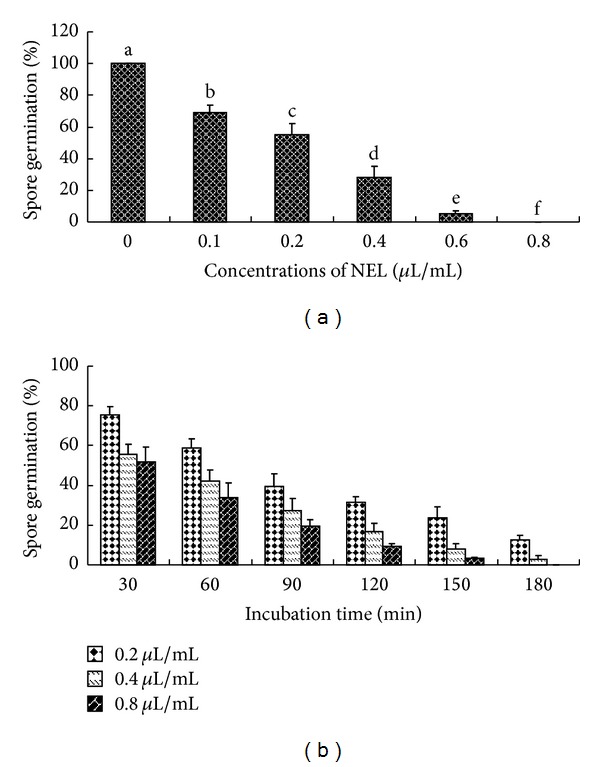
Effects of NEL on spore germination of *A. flavus*. (a) Effects of different concentrations of NEL on spore germination of tested fungi. (b) Growth kinetics of the inhibition of the tested fungi by NEL.

Results in Figure 3 present the effects of NEL on spore germination of *A. flavus*.  Figure 3(a) indicates that reduction in percentage of spore germination is evident (*P* < 0.05) as NEL concentration increased. All* A. flavus* spores germinated after 20 h of incubation at 28°C in PDB without the NEL. However, the highest concentration (0.8 *μ*L/mL) of NEL caused most profound reduction of spore germination. Observations revealed inhibitory effects on the spore germination of *A. flavus* within the range of 5.3–69.3% at concentrations ranging from 0.1 to 0.6 *μ*L/mL.

The inhibition growth kinetics of *A. flavus* by the NEL is exhibited in Figure 3(b). Exposure of the *A. flavus* spores to different concentrations of the NEL for a period of 0–180 min caused varying degrees of spore germination inhibition. The results of the present investigation emphasize that the NEL was effective inhibition on spore germination at varying concentrations and there was a positive correlation between NEL concentrations and exposure period. The NEL at 0.2 and 0.4 *μ*L/mL showed antifungal activity but not rapid killing and about 20–40% inhibition was observed at an exposure time of 90 min. However, there was a visible increase in the killing rate at 0.8 *μ*L/mL after 30 min of exposure and complete inhibition of spore germination was observed at 180 min of exposure.

### 3.3. Antifungal Effects of NEL on Dry Mycelium Weight and AFB_**1**_ Content

**Figure 4:**
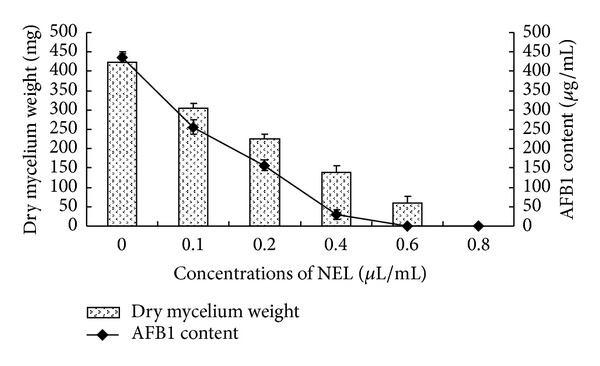
Effects of the different concentrations of NEL on dry mycelium weight and AFB_1_ production by *A. flavus. *Values are mean (*n* = 3) ± standard deviations.

Figure 4 exhibits the effects of NEL on *A. flavus* aflatoxin synthesis and mycelial dry weight in PDB medium. All treatments consisting of the five different concentrations of NEL effectively caused different degrees of inhibition of dry mycelium weight and AFB_1_ synthesis (*P* < 0.05). The results indicated that NEL strongly inhibited AFB_1_ production in a dose-dependent manner. NEL totally inhibited the mycelial production at 0.8 *μ*L/mL. However, mycelial growth was observed at 0.6 *μ*L/mL, although AFB_1_ production was completely inhibited. The NEL caused an apparent inhibition in AFB_1_ content at 0.1, 0.2, 0.4, and 0.6 *μ*L/mL concentrations with inhibition percentages of 41.6%, 64.0%, 93.3%, and 100.0%, respectively (data not shown).

### 3.4. Effects of NEL in the Conservation of Cherry Tomatoes

**Figure 5:**
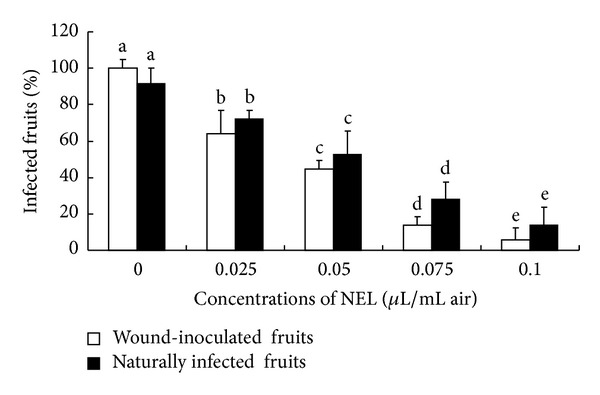
Effects of NEL on fungal development in wound-inoculated cherry tomatoes and naturally infected fruits. Significant differences (*P* < 0.05) between means are indicated by letters above the histogram bars. Values are mean (*n* = 3) ± standard deviations.

The presented results (Figure 5) show the *in vivo* effect of NEL can control fungal development in wound-inoculated cherry tomatoes caused by *A. flavus *and natural decay development in unwounded cherry tomatoes. The artificial decay incidence of the control groups for *A. flavus* was 100% after 9 days of storage at 18°C. It was found that the percentages of infected fruits were significantly (*P* < 0.05) reduced in the treatment group compared with the control group and also significantly (*P* < 0.05) reduced with increasing concentrations of NEL. The most effective concentration of 0.1 *μ*L/mL air produced the greatest reduction in the percentages of decayed fruits for *A. flavus* compared with that of the control group, with percentage reductions of 94.4%. After 21 days of storage at 18°C, the results revealed that the percentage of infected fruits was significantly (*P* < 0.05) reduced by NEL. In this case, the concentration of 0.1 *μ*L/mL air exhibited the highest inhibition of fungal development compared with that of the control group, at 77.8%. As shown in Figure 5, the protective effect of NEL on artificially inoculated fruits is obviously higher than naturally infected healthy fruits after NEL vapor treatment.

### 3.5. Effects on the Sensory Characteristics of Fruits

The overall acceptability scores of cherry tomatoes as affected by NEL are summarized in Table 1. As presented in Table 1, no significant effects were seen in NEL-treated fruits except for the highest concentration at 7 days of storage in naturally infected groups. Overall, scores for NEL-treated fruits and the controls were considerably increased with storage time and the increasing concentration of NEL. The concentration of 0.1 *μ*L/mL air indicated significantly higher scores compared with that of the control in both wound-inoculated and naturally infected fruits.

## 4. Discussion

Today, special attention has been paid to seek natural, safe, and effective food preservatives to control contamination and the growth of spoiling fungi. NEL has a sweet rose odor and is originally from essential oil which are classified as “generally regarded as safe” (GRAS) by the United State Food and Drug Administration (FDA) [15]. Some acute toxicity tests for oral, dermal, and intramuscular have proved that NEL is very safe, for example, the acute oral LD50 of NEL was evaluated to be 4.5 g/kg by Wistar rats [5]. Moreover, NEL are abundant and its use worldwide is in the region of 100–1000 metric tonnes per annum [5], which makes NEL suitable for development into a natural food preservative.

In our present work, mycelium growth was recorded to change with increasing concentrations of the NEL and incubation time, and the colony diameter expanded along with the increase in the number of incubation days. The results are consistent with previous findings by Avila-Sosa et al. [16]. However, the results reported in the present study indicate that the vapor phase of NEL results in higher inhibition than the contact phase to the tested fungi *in vitro*. Some investigators revealed that antifungal activity results from a direct effect of terpenes vapors on fungal mycelium, and they postulated that the lipophilic nature of terpenes allow them to be absorbed into fungal mycelia [17].

The spore is another important structure for the survival and spread of fungi [18]. NEL was also efficacious inhibiting the germination of *A. flavus*. Reduction in percentage of spore germination increasement is evident as NEL concentration increased. The effects of some compounds on fungal spore germination were also conducted. Yenjit et al. [19] found that fernenol, arundoin, and a mixture of stigmasterol and *β*-sitosterol greatly inhibited spore germination and germ tube elongation in *Colletotrichum gloeosporioides* with EC_50_ values of 45.8, 62.3, and 86.9 mg/L. Recently, Nishino et al. [20] showed that 1-Phenyl-3-pentanone from an edible mushroom had significantly inhibitory spore germination of some plant-pathogenic fungi against plant-pathogenic fungi at 35 ppm. As presented in Figure 3, the kinetic study of *A. flavus *revealed that the exposure time of the NEL had a little effect on the antifungal activity at 0.2 *μ*L/mL. However, spore germination was considerably restrained at 0.4 and 0.8 *μ*L/mL. Similar results of reduced spore germination effect was observed by Bajpai et al. [21]. The antifungal mechanism of terpenes could be involved in its ability to disrupt the permeability barrier of the plasma membrane and the mitochondrial dysfunction-induced ROS accumulation in *A. flavus *[22].

AFB_1_, produced by *A. flavus*, is the most dangerous toxic metabolite among all classes of aflatoxin. Therefore, a measurement of the content of AFB_1_ was conducted in the present study. Based on the present study, the NEL showed a remarkable effect in restraining dry mycelium weight and the AFB_1_ production of *A. flavus*. The NEL indicated antiaflatoxigenic properties at concentrations lower than its fungitoxic concentration; a similar supportive study was also performed by Shukla et al. [23] and Prakash et al. [24]. This could be related to different modes of actions of the NEL against fungal growth and aflatoxin production. Several research workers have demonstrated that there is a direct correlation between fungal growth and AFB_1_ production [10, 25]. Nevertheless, the inhibition of AFB_1_ synthesis cannot be completely attributed to the insufficient fungal growth, but it may be a result of the inhibition of carbohydrate catabolism in *A. flavus* by acting on some key enzymes leading to the reduction of its ability to produce AFB_1_ [26]. No exact mechanism of AFB_1_ synthesis of terpenoids has been available in the scientific literature until now. In light of our results, it could be concluded that the NEL may interfere with some steps in the metabolic pathways of the *A. flavus*, which controls the biosynthesis of AFB_1_. Thus, further experiments on NEL are required to understand the AFB_1_ suppression mechanisms.

Cherry tomatoes are generally susceptible to various pathogens such as *A. flavus* due to the warm and humid climate in which it grows and have a short shelf life [27]. Data obtained from *in vitro* experiments on NEL disclose its potential as an ideal antifungal agent against *A. flavus*. Thereby, further *in vivo* tests on its efficacy as a natural preservative for the control of rotting in fruit is necessary. Under *in vivo* conditions, the NEL possessed strong antifungal activity when it was applied to cherry tomatoes artificially contaminated with *A. flavus*. NEL was also effective against the native fungal microbiota of fruits. Indeed, the use of vapor treatments is ideal for controlling food spoilage because it leaves no residual components [28]. Our results are in agreement with the previous findings of Perez-Alfonso et al. [29] who reported thymol, carvacrol, and the mixture of both pure essential oils controlled lemon spoilage pathogens. Our *in vivo* trial indicates that exposure of produce to the vapors may induce higher resistance to fungal challenges. Inhibitory effects of the NEL on fungi observed under vapor treatments could be due to hydroxyl groups in antimicrobial compounds forming hydrogen bonds with active enzymes resulting in deactivation [30].

In the panel test, a sensory analysis was conducted to measure the viability of the NEL using concentrations determined. The control presented a very obvious decrease in visual appearance and had the lowest scores, to the extent of being unacceptable for consumers, which could be attributed to the high decay incidence and the change in carbohydrates, proteins, amino acids, lipids, and phenolic compounds that can influence the fresh fruits [31]. Nevertheless, the treatment of fruit with NEL had higher scores from the presented data. As was expected, the application of NEL as a promising food preservative improved the sensory aspects of overall acceptability throughout storage period. In agreement with the present findings, other studies have reported terpenes can help in maintaining quality of fruits [32–35].

## 5. Conclusion

The present results of *in vitro* trials pointed out that pronounced antifungal properties of NEL against *A. flavus* related to its activity to degeneration of mycelial growth, spore germination, and AFB_1_ synthesis. Moreover, *in vivo* studies also indicate that NEL can produce growth inhibitory effects against *A. flavus* and a broad spectrum of fungal microbiota of cherry tomatoes. Employing NEL as a significant fumigant during routine storage or extended transport is very promising. The use of NEL cannot only improve food safety by eliminating fungal spread but also leaves no detectable residues after storage. Therefore, NEL would be economical, with considerable commercial significance, and is worthy of further investigation when used as fumigant in storage containers.

## Figures and Tables

**Table 1:** Mean scores of overall acceptability of stored cherry tomatoes treated with NEL.

Conc. of NEL (*μ*L/mL air)	Wound-inoculated fruits			Naturally infected fruits		
	Day 3	Day 6	Day 9	Day 7	Day 14	Day 21
Control	6.83 ± 0.93^a^	3.17 ± 0.72^a^	1.17 ± 0.39^a^	7.08 ± 1.00^a^	3.33 ± 1.43^a^	1.08 ± 0.29^a^
0.025	7.17 ± 1.11^ab^	3.92 ± 0.90^ab^	2.67 ± 0.89^b^	7.25 ± 0.87^a^	5.17 ± 1.34^b^	2.50 ± 0.80^b^
0.05	7.58 ± 1.08^abc^	4.17 ± 1.27^b^	3.17 ± 1.19^b^	7.58 ± 1.00^ab^	5.92 ± 1.24^b^	3.91 ± 1.00^c^
0.075	7.92 ± 0.90^bc^	5.83 ± 1.11^c^	4.92 ± 1.24^c^	7.75 ± 0.87^ab^	6.25 ± 1.22^b^	5.42 ± 1.31^d^
0.1	8.17 ± 0.94^c^	7.42 ± 1.08^d^	6.25 ± 0.87^d^	8.25 ± 0.97^b^	7.58 ± 1.38^c^	7.33 ± 1.07^e^

Conc.: Concentration.

^a∼e^Values are mean (*n* = 3) ± standard deviations. Values followed by the same letter in each column are not significantly different in ANOVA and Duncan multiple range test (*P* < 0.05).
